# COVID-19 Vaccination Messengers, Communication Channels, and Messages Trusted Among Black Communities in the USA: a Review

**DOI:** 10.1007/s40615-023-01858-1

**Published:** 2023-11-10

**Authors:** Yael Rabin, Racquel E. Kohler

**Affiliations:** 1 Department of Health Behavior Society and Policy, Rutgers School of Public Health, Piscataway, NJ USA; 2 Center for Cancer Health Equity, Rutgers Cancer Institute of New Jersey, New Brunswick, NJ USA

**Keywords:** COVID-19 vaccination, Health communication, Communication inequalities, Vaccine hesitancy, Systematic review

## Abstract

Black and African American adults exhibited higher levels of mistrust and vaccine hesitancy and lower levels of vaccination throughout the COVID-19 pandemic. Vaccination and booster uptake remains disproportionately low among Black adults. We conducted a systematic review of empirical research published between February 2021 and July 2022 from five electronic databases and the grey literature. We screened studies that assessed COVID-19 vaccination information needs and preferences as well as communication strategies among Black adults in the USA. We extracted data, then analyzed and synthesized results narratively. Twenty-two articles were included: 2 interventions, 3 experimental surveys, 7 observational surveys, 8 qualitative inquiries, and 2 mixed methods studies. Studies reported credible and preferred COVID-19 vaccination information sources/messengers, channels, and content. Commonly trusted messengers included personal health care providers, social network connections, and church/faith leaders. Electronic outreach (e.g., email, text messages), community events (e.g., forums, canvassing), and social media were popular. Black communities wanted hopeful, fact-based messages that address racism and mistrust; persuasive messages using collective appeals about protecting others may be more influential in changing behavior. Future communication strategies aiming to increase vaccine confidence and encourage COVID-19 booster vaccination among Black communities should be developed in partnership with community leaders and local health care providers to disseminate trauma-informed messages with transparent facts and collective action appeals across multiple in-person and electronic channels.

**Supplementary Information:**

The online version contains supplementary material available at 10.1007/s40615-023-01858-1.

## Introduction

Black or African Americans have been disproportionally impacted by COVID-19 with higher hospitalization and death rates compared to white Americans [1]. COVID-19 outcomes have been exacerbated by low vaccination uptake and comorbidities. Initial vaccination campaigns in April 2021 documented a 14-percentage point gap between Black and White Americans. Although the gap was reduced by July 2022, fewer Black adults had received at least one dose compared to White adults [2]. However, COVID-19 boosters have widened the gap again: 21.2% of Black compared to 32.1% of White adults have received the bivalent COVID-19 booster shot as of December 2022 [3]. Vaccination rates have fluctuated since their authorization and approval, but hesitancy has been pervasive [4, 5]. The determinants of hesitancy vary by communities and socio-cultural factors including race/ethnicity, therefore requiring a nuanced approach to public health campaigns.

In Black communities, vaccine hesitancy and medical mistrust stem from a dark history of unethical medical experiments and persist with racism and mistreatment contributing to health inequities. Structural barriers and communication inequalities left many low-income and low-literate communities with limited access to COVID-19 information which contributed to lower rates of vaccination [6]. Developing a deeper understanding of how to increase vaccine confidence in Black communities through communication strategies is critical to support policy and programs promoting COVID-19 vaccination and may provide helpful insights into future public health campaigns.

There is a growing body of literature exploring attitudes and perspectives of COVID-19 vaccine hesitancy, information sources, and communication preferences among specific geographic, racial/ethnic, and cultural communities. One narrative review reported on recommendations for vaccine uptake [7]; however, they did not exclusively include empirical data in their review and did not examine strategies and approaches used to promote COVID-19 vaccination. The purpose of this systematic review is to investigate empirical evidence of credible, trusted, and preferred health messengers, messages, and communication channels regarding COVID-19 vaccination among Black adults to inform ongoing and future vaccination and booster interventions with Black communities as the priority audience. Specifically, this review aims to identify the appropriate health messengers to promote vaccine confidence and message content that address vaccine hesitancy among Black communities in the USA, and which communication channels they prefer.

## Methods

We conducted a systematic literature review to analyze findings from empirical studies in peer-reviewed and grey literature. We focused on studies that collected and analyzed quantitative and/or qualitative data from Black adults in the USA on topics including communication strategies and information needs related to COVID-19 vaccination. Literature searches occurred between June and August 2022 to assess literature written and published 6–8 months after the COVID-19 vaccine first became available in December 2021. This review included articles published between February 2021 and July 2022.

### Search Strategy

We searched PubMed, World of Science, CINAHL, Academic Search Premier, and ProQuest Social Science Database. Key terms were identified and applied in the same methodical pattern within each search engine. The first and second term in each search string was “COVID-19 vaccine OR COVID-19 Vaccination” and “African American OR American black OR Black,” respectively. The third search term alternated among “Health Information,” “Health Message,” “Trusted Sources,” “Social Networks,” “Information Sources,” “Persuasive Communication,” and “Community-based” in each database. Note that the use of the terms “African Americans,” “Blacks,” “American blacks,” and “Black Americans” varied among the articles.

### Eligibility Criteria

This study followed the Preferred Reporting Items for Systematic Reviews and Meta-Analyses (PRISMA) recommendations [8]; the review protocol was not registered. The inclusion criteria for this review required that the studies report on interpersonal or organizational information sources and communication strategy preferences (e.g., message content, framing, channels) among Black adults in the USA. We included English-language studies that enrolled solely Black participants, reported 10% of the full sample identified as Black, African American, or Black immigrant, or reported results by race/ethnicity groups. Empirical research that explored COVID-19 vaccination beliefs or behaviors or tested communication strategies were included. Studies were excluded if they focused on participants with underlying health conditions, were based in a hospital or institution, were published prior to 2021 when the vaccine became first became available, or took place in or analyzed data from countries outside of the USA. We did not include conference abstracts, commentaries, editorials, scoping, or systematic reviews.

### Study Selection

After duplicates were removed, search results were exported into EndNote for title/abstract and full-text screening. A manual review of relevant excluded literature reviews and commentaries identified two additional studies. Articles were included by agreement with both authors after full-text screening using the eligibility criteria. Disagreements were discussed and resolved to ensure they represented this review’s criteria. The quality of the studies and appropriateness of the methods and techniques (e.g., design, sampling, data collection, analytic methods, stakeholder considerations) were assessed according to the Quality Appraisal for Diverse Studies (QuADS) tool, which allows for assessments of multiple and mixed methods research study designs [9]. Criteria were agreed upon, applied, compared, and discussed by the authors.

### Analysis and Reporting

Data were extracted from each study and analyzed using a narrative/evidence synthesis. A meta-analysis was not feasible due to the heterogeneity of study designs and outcomes. We extracted and described study characteristics and grouped them by geographic location and sub-populations. We extracted behaviors, intentions, communication outcomes, or inputs that were significant in quantitative studies and described aspects of the communication process: source/messenger (credibility or trustworthiness), channels (modality), and message (style or content) and thematically analyzed results from qualitative studies. When possible, we used the original study’s language to differentiate between populations (e.g., non-Hispanic Black, Black Latinx, African Americans, African immigrants), but due to varying means of collecting and reporting ethno-racial identity, we generally describe the results in terms of Black individuals or communities.

## Results

### Search Outcomes

**Fig. 1:**
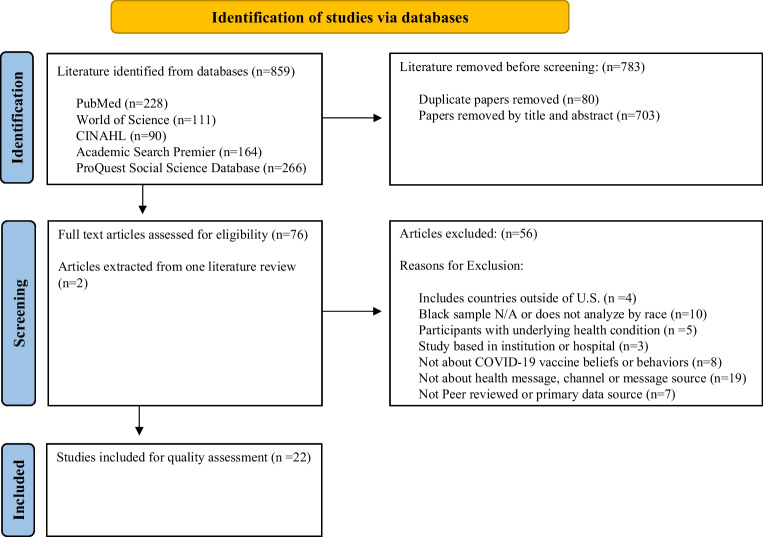
PRISMA flow diagram of search results and study selection

A total of 859 papers were identified and after removing duplicates, 779 were retained for title/abstract screening. Seventy-eight articles underwent full-text screening, and 56 were excluded from the analysis after critical full-text review as they did not meet the inclusion criteria. Twenty-two were deemed eligible and data, were abstracted independently by both study authors. After reviewing QuADs scores (Supplementary Table), we retained all studies despite some lower scoring criterion (e.g., experiments and surveys scored low on stakeholder considerations) as there is no threshold score for high or low quality [9]. Details of the screening process and reasons for exclusion are reported in Fig. 1.

### Study Characteristics

Twenty-two studies analyzing trusted message sources, health messages, and/or communication channels for COVID-19 vaccination were included in this review (Table 1). The types of studies included two intervention trial or implementation evaluations (non-randomized study of intervention) [10, 11], three experimental surveys [12–14], seven observational surveys [15–21], eight qualitative methods (i.e., interviews or focus groups) [22–29], and two mixed methods studies [30, 31]. Five studies enrolled non-Hispanic Black or African American participants exclusively [14, 15, 18, 24, 28] with the remaining studies’ sample compositions of Black participants ranging from 12 to 95%. We identified multiple sub-populations including older adults aged ≥ 65 years, young adults aged 18–30 years, safety-net patients, pediatric patients and families, pregnant/postpartum women, barbershop owners and workers, and people affiliated with faith-based organizations. Nine study settings were specific cities or metro areas (i.e., Boston, Cincinnati, Denver, New Haven, New York City, Philadelphia, San Francisco, Shreveport, Washington, D.C.); three were across states or regions (i.e., California, central Missouri, Kansas); ten recruited across the entire USA.

**Table 1:** Descriptions and characteristics of studies for review

	Study objective, research question	Data collection, methods, analysis	Study time period	Study population	Sample size	Proportion of sample identifying as Black/African American	Study setting
**Intervention or implementation study**							
Burkhardt et al. (2022)	To evaluate implementation of universal model offering COVID-19 vaccines to pediatric patients and household members during routine visits	Descriptive implementation; electronic health records (EHR)	May–Nov 2021	Unvaccinated pediatric patients and household members	746 patients, 630 adults	64.1%	3 pediatric practices affiliated with Cincinnati Children’s Hospital Medical Center in Ohio
Lieu et al. (2022)	To evaluate vaccination after electronic/mail outreach from Primary Care Physicians (culturally tailored or standard) compared to usual care	3-arm randomized trial; Kaiser Permanente Northern California (KPNC) EHR and state immunization data	March–July 2021	Unvaccinated Black and Latino older adults aged ≥ 65 years	8287	29.4%	4 KPNC service areas: California Central Valley, Fresno, South Sacramento, and San Jose
**Experimental survey**							
Dhanani and Franz (2022)	To examine effects of 3 messaging strategies (general information, social justice, ethical oversight) on willingness to vaccinate and vaccine hesitancy compared to no information	Experiment stratified by race with random assignment to one of four messages	April 2021	Unvaccinated Black, White, Hispanic adults	743	32.8%	National web-based survey panel (Prolific Academic)
Gadarian et al. (2022)	To test whether COVID-19 vaccination information from same-race/ethnicity experts increases vaccination intention, information seeking, and information sharing	Experiment with random assignment to message from the same or different race/ethnicity experts	March–April 2021	Oversamples of unvaccinated Black, Hispanic, and Asian American adults	2117	22.2%	National web-based panel (YouGov)
Huang and Green (2022)	To determine if a self-persuasion narrative is more effective to elicit positive vaccination beliefs and intention than self-persuasion task, a non-narrative message, or control group	Experiment with random assignment to pro-vaccine messages or self-persuasion task	June 2021	Unvaccinated African Americans (AA)	394	100%	National web-based panel (Qualtrics)
**Observational survey**							
Bogart et al. (2021)	To understand the factors contributing to COVID-19 vaccination intentions	Online survey	Nov–Dec 2020	Unvaccinated Black adults	207	100%	National web-based panel (RAND America Life Panel)
Davis et al. (2022)	To examine COVID-19 vaccine concerns, acceptance, and trusted information sources	Interviewer- administered phone survey	Feb–Oct 2021	Safety-net health system patients with recent outpatient visit	204	52%	Shreveport Louisiana
Fisher et al. (2021)	To determine whether patient preferences for being informed about the COVID-19 vaccine and where to receive it are associated with vaccination intent and race/ethnicity	Online survey	Jan–Feb 2021	Unvaccinated adults, with oversample of Black and Latino adults	1668	33.3%	National web-based survey panel (Prolific Academic)
Francis et al. (2021)	To examine relationships between family communication and COVID-19 vaccination attitudes and intention	Online survey	June 2020	Young Black/AA adults aged 18–30 years	312	100%	National web-based panel (Qualtrics)
Karpman et al. (2021)	To examine how vaccine concerns, trust in community sources of information, and connections to the health care system vary by race, ethnicity, and political party affiliation	Online survey (Urban Institute Well-Being and Basic Needs Survey) with open-ended questions	Dec 2020	Oversample of low-income households of adults aged 18–64	7737		National web-based panel (Knowledge Panel)
Kricorian and Turner (2021)	To explore COVID-19 vaccination willingness, delay, and mistrust and examine factors to address hesitancy	Online survey	Jan 2021	Unvaccinated adults	1950	12%	National web-based panel
Redmond et al. (2022)	To investigate vaccination perspectives and decision-making	Online survey with open-ended questions	June–Aug 2020	Pregnant or postpartum women	27	66.7%	Kansas
**Qualitative inquiry**							
Balasuriya et al. (2021)	To investigate factors that facilitate and prevent COVID-19 vaccine acceptance	8 online focus groups	March–July 2021	Black and Latinx communities	72	50% Black, 11% Black Latinx	Community-based organizations (CBO) and community health centers in New Haven, Connecticut
Butler et al. (2022)	To explore knowledge and beliefs about COVID-19 vaccination and assess perspectives of outreach and delivery strategies	10 online focus groups and 25 in-depth interviews	Jan–Feb 2021	Black/AA, Latinx, and Chinese adults	109	32.1% (from focus groups)	3 CBOs in San Francisco Bay area
Dong et al. (2022)	To examine intentions and beliefs as well as trusted sources and suggestions for information campaigns	Semi-structured phone interviews	Dec 2020–March 2021	Black adults with low/no vaccination intention	24	100%	Subgroup of national web-based panel (RAND American Life Panel) survey respondents
Majee et al. (2022)	To explore vaccination intentions and attitudes and the role of a faith-based wellness program in COVID-19 vaccination uptake	Phone and in-person in-depth interviews	April 2021	AA faith-based wellness program participants, coaches, leaders	21	95%	Central Missouri
Momplaisir et al. (2021)	To understand beliefs, attitudes, and norms regarding COVID-19 vaccines	4 virtual focus groups	July–August 2020	Black barbershop, salon owners	24	89%	West Philadelphia
Osakwe et al. (2021)	To understand barriers and facilitators of COVID-19 vaccine acceptance	Virtual, phone, and in-person semi-structured interviews	Feb–March 2021	Black and Hispanic adults	50	68% non-Hispanic Black, 6% Black Hispanic	New York metropolitan area
Sekimitsu et al. (2022)	To determine hesitancy, motivators, and trusted information sources of COVID-19 vaccination	Virtual semi-structured interviews	March–April 2021	Vaccine hesitant Black adults involved with African Methodist Episcopal Church	18	100%	Boston, MA
Zhou et al. (2022)	To explore barriers and facilitators to COVID-19 vaccination, acceptability of chatbots delivering information, and preferences for culturally tailored messages	Virtual semi-structured interviews	Oct–Nov 2020	Hispanic/Latino American, Black/AA, and American Indian/Alaska Native adults	18	44.4%	Denver Metropolitan area
**Mixed methods**							
Kerrigan et al. (2022)	To explore barriers and facilitators to COVID-19 vaccination and to inform COVID-19 vaccine awareness strategies	Online interviews, 5 focus groups, and crowdsourced voting	Dec 2020–Feb 2021 interviews, March 2021 focus groups, May–July 2022 voting	Black AA, African Immigrant, and Latinx adults	40 interviews, 36 in focus groups, 208 voting	46% AA, 16% African immigrant	3 CBOs and department of health in Washington D.C
Lee Rogers and Powe (2022)	To identify the receptivity to and spread of COVID-19 misinformation by faith communities and willingness to use face masks and get vaccinated	Automated text mining of online discussion forums/social media posts and online surveys	Jan 2020–Nov 2021 forums; April 2020 and Feb 2021 surveys	Religious COVID-19 posts; unvaccinated adults	328,638posts; 9482 in 2020; 9419 in 2021	2020 survey: 11% Black; 2021 survey: 12% Black Protestants	91 discussion forums/chat rooms; national web-based panel (Pew American Trends Panels)

### Health Messengers

Most (18) studies reported on health messengers and/or information sources. We identified nine key categories of interpersonal and organizational messengers that were commonly relied upon, considered credible, or described as trustworthy (Table 2).

**Table 2:** Trusted messengers for the COVID-19 vaccination

Trusted messenger	Number of endorsing studies
Primary care/personal physician	12
Social network (e.g., friends, family, neighbors)	9
Church and faith leaders	7
Local and/or state government officials (e.g., Department of Health)	6
Other health care professionals (e.g., nurses, pharmacists, clinic staff)	5
Community leaders, community-based organizations	5
Celebrities, public figures, politicians	4
Scientists, researchers, public health experts	3
Federal health agency (e.g., CDC/FDA)	1
*Total number of studies reporting on trusted messengers*	*18*

Personal physicians were mentioned in more studies than any other type of messenger and physician consultations or recommendations were associated with vaccination [10, 11, 16, 17, 19–21, 23, 24, 26–28, 30]. Other health care professionals and professional associations, including nurses, pharmacists, and community health clinic staff, were also mentioned as trustworthy messengers in multiple studies [16, 21–23, 30]. A few studies found that Black health care providers, scientists, and researchers enhanced trustworthiness [24, 27, 30] and may increase acceptance of vaccination [20]. However, one experimental survey found messages from experts with ethno-racial identity concordant to the receiver had no effect on vaccine intentions [13]. Another survey emphasized pregnant/postpartum women would be reassured if they knew their recommending provider was vaccinated [21] and recommendations from pediatricians were sometimes cited as influential in decision-making among family members of pediatric patients [10].

People in social networks including friends, family members, neighbors, and peers were the second most reported messenger across all studies [10, 16, 18, 22, 25, 27, 28, 30, 31]. Notably, input from family members was a common reason for acceptance [10]. Social/family vaccination norms and interpersonal communication were linked with positive vaccination intentions [15, 18] partly by facilitating a return to in-person activities [28].

Church and faith leaders were the third most common messengers reported across studies [16, 22, 23, 25, 28–30]. Ministers, faith groups, and other leaders at houses of worship were important COVID-19 information messengers across multiple groups with different ethno-racial identities, but they were particularly emphasized as trustworthy among African Americans and African immigrants. Similarly, community leaders and other community-based organizations (CBOs) [22, 23, 25, 27, 30] were also trusted. Trusted CBOs were often linked with providing access to the vaccine, typically at well-known locations including community clinics, centers, churches, or schools.

Findings about the influence of celebrities and public figures were mixed. Two studies reported that promoting celebrities or famous politicians modeling vaccination was not persuasive [26, 28], but participants relied on some public figures and experts (e.g., Dr. Anthony Fauci) for vaccination information. Still, two others noted African American participants would like to see celebrities [24] and government officials get vaccinated [29]. One survey found African American adults’ willingness to get vaccinated was more likely to increase with endorsements from celebrities than adults of other ethno-racial identities [20]. However, multiple studies reported representation of community members or public figures in vaccine messaging was important to participants with different ethno-racial identities (e.g., Hispanic/Latinx, Chinese American) [22, 23, 27, 30].

A few studies noted that Black adults were more likely than non-Hispanic White and Hispanic/Latinx adults to consider federal agencies (i.e., CDC, FDA), local or state governments (e.g., departments of health) [16, 28, 29] and/or public health officials [19, 31] credible or trustworthy information sources. However, more studies reported on mistrust of government as described below.

### Communication Channels

Two studies evaluated vaccination outcomes in practices or health care systems after interventions. Burkhardt and colleagues reported over 70% of eligible pediatric patients and household members completed two doses after in-person counseling/recommendations from a pediatric provider [10]; however, Black household members were less likely to vaccinate compared to White individuals. Lieu and colleagues found that electronic messages and follow-up postcards from patients’ primary care providers increased vaccination among older adults compared to usual care (no outreach), but they did not evaluate differences by outreach attempts or channel [11].

Nine other studies reported on perspectives about commonly used communication channels and/or future preferences for how African American individuals would like to learn about COVID-19 vaccination. We identified seven categories from the findings (Table 3). Electronic outreach, usually in the form of emails, text messages, and health portal messages, was commonly valued, with one study reporting about African Americans’ reliance on neighborhood/community listservs and newsletters [31]. Online and in-person community events or forums were suggested [21, 24] in addition to door-to-door canvassing as ways to help increase access to information [27, 30] and give opportunities to ask questions. Telephone hotline numbers or chatbots were also strongly desired because of the real-time, interactive nature of getting answers to questions immediately [29, 30]. Face-to-face and telephone conversations with a health care provider were specifically important among individuals with low intentions and pregnant and postpartum women [17, 21].

**Table 3:** Preferred communication channels for COVID-19 vaccination

Communication channel	Number of endorsing studies
Electronic outreach (e.g., email, text, portal messages)	4
Community outreach and events (e.g., canvassing, forums, presentations)	4
Social media	4
Traditional media (e.g., TV, radio)	3
Direct conversation or phone call with health care provider	2
Question/Answer telephone hotline, chatbot	2
Printed materials (e.g., mailers, flyers)	2
*Total number of studies reporting on communication channels*	*9*

Although many of these channels were generally suggested as acceptable by the larger Black community, we found some differences by sample sub-populations. Print materials, such as flyers and pamphlets, were suggested specifically for older adults [17, 30]. Traditional media was suggested for widespread reach [25, 30], but some studies indicated these were less trustworthy channels [16] and multimedia platforms were important [27]. Social media was also recommended, though mainly to reach younger adults [21, 24, 27, 30].

### Message Features

Three studies evaluated different aspects of messages (e.g., content, style, and framing) using a randomized trial [11] or experimental survey [12, 14]. The study populations were predominately non-Hispanic Black, Hispanic, and/or Asian Americans; however, the outcomes (e.g., vaccination intention, vaccine hesitancy, and vaccination) and the control/usual care intervention varied. We report the findings among non-Hispanic Black participants. Dhanani and Franz found that messages acknowledging past medical harm and current mistreatment precautions were linked to lower vaccine hesitancy among Black participants [12]. Huang and Green found that self-persuasion narratives elicited greater vaccination intentions among African Americans compared to a plain narrative and a self-persuasion task, particularly among participants with low trust in science [14]. Lieu and colleagues found that standard vaccination messages and culturally tailored messages addressing cost and access barriers both increased vaccination among older Black adults [11].

Multiple survey and qualitative studies reported on preferred message types and communication styles including highly valued COVID-19 vaccination information/content and framing. Half of the studies described safety concerns about the new vaccines and development timeline, so not surprisingly they largely described African American participants wanting to wait and see more data [26, 29]. Detailed content about the development process and safety results were desired [20, 28], especially about Black/African American trial participants, pregnant people [21], and individuals with pre-existing conditions [25]. Generally, studies indicated that content should be based on facts and evidence with transparent and honest messaging. Including clinical trial findings about immediate and potential long-term side effects was important to address safety concerns [22, 24, 27–29].

Positive, motivational, and hopeful messages resonated more than fear-based appeals [23, 25, 29, 30]. A few studies also noted framing vaccination as a choice instead of an obligation or mandate would be more effective [22, 30] and may promote more control and freedom to resume social and work activities, which was highly valued among African American participants [23, 24, 28, 29]. Persuasive messages were notably community-focused or appealed to collective action: participants in two studies responded positively to messages about protecting family members, especially children and the elderly, as well as the “social other” because they elicited a sense of unity [23, 29].

Mistrust or low levels of trust in government, health care systems, pharmaceutical companies, and vaccines in general were reported in thirteen studies [15, 16, 19, 20, 22–30]. While some surveys found associations between mistrust, low confidence, high hesitancy, and low intentions, many qualitative studies explored reasons for COVID-19 vaccine hesitancy. The findings were mixed regarding *how* historical racism and mistreatment should be acknowledged; however, most described that messages addressing mistrust and systemic injustices were needed, wanted, and may help build trust among African Americans [23–25, 27, 30].

## Discussion

This review of 22 empirical studies on COVID-19 vaccination communication strategies summarized trusted messengers, preferred communication channels, and important message characteristics identified by Black and African American adults. The most trustworthy messengers across the studies we reviewed were personal connections or those with shared community and/or identity: personal physicians/primary care providers, social network contacts, and church/faith leaders. Communication channels differed somewhat by audience segments, mainly age-specific preferences. Safety concerns and mistrust influenced hesitancy, so transparent messages with facts that recognize past and current experiences of the Black community were desired.

We found that trusted messengers were individuals who had longstanding, personal relationships with community members. Similar to the uptake of other vaccines [32, 33], a primary care physician or personal health care provider was the most commonly identified trusted messenger for COVID-19 vaccination across the studies we reviewed. These findings demonstrate the importance of including primary care providers in vaccination outreach and education and additional resources for providers to address misinformation and concerns during counseling conversations [34, 35]. Although the influence of a physician recommendation on vaccination is consistent across other ethno-racial identity groups and other vaccines [36–38], some studies emphasized that Black health care providers, researchers, and scientists may increase acceptance among Black community members. Indeed, others have suggested that Black pharmacists promoting and administering the COVID-19 vaccine may be important to help expand access and foster trust in Black communities [39]. Therefore, communication strategies should ensure Black health care providers, ideally from the local area, are messengers of COVID-19 vaccination information as representation may increase vaccine confidence in the Black community.

Continuing with the finding that trusted messengers are often personal connections or someone embedded in the local community, contacts within social networks, faith and community leaders, and local and state government officials were also trusted. In some studies, information from and vaccination modeling by these messengers were associated with positive vaccine intentions. This finding is similar to those of other vaccines (e.g., HPV, flu) where community-based organizations and social peers are trusted and can influence uptake [40, 41]. For example, social norms and family culture were particularly influential on flu vaccination among African American adults [38]. Because of strong social ties and network connections, locally driven vaccination campaigns in Black communities should engage trusted leaders with shared identities of the community, including pastors and health officials, to help plan, implement, and disseminate normative messages about vaccination [42]. Social network campaigns or interventions that encourage friends and family to share the benefits of vaccination may accelerate vaccination behavior change by reaching people in their own communities [43, 44].

Preferences for communication channels ranged from digital and traditional media to direct communication in person or over the phone. Though electronic outreach was common, printed materials were preferred among older adults. Social media, though acceptable for the general population, was particularly suggested for reaching younger generations, which is consistent with promotion efforts for other vaccines [45, 46]. A desire for communicating through trusted community channels was reflected in preferences for outreach events and meetings, Q&A sessions, and community canvassing. Indeed, community-based communication to promote COVID-19 vaccination through CBO-sponsored events like virtual town halls and in-person tabling at community fairs has been useful in reaching groups with other ethno-racial identities [47–50]. Speaking directly with a primary care provider—both face-to-face and over the phone conversations—was another important mode of communication. Direct conversations may be key opportunities to establish trustworthiness, acknowledge historical and current harmful experiences, and build trust among hesitant African American patients [51–53]. Recognizing the diversity of Black communities across the USA, these findings highlight the importance of partnering with CBOs to deploy a multimodal approach using different communication channels appropriate for reaching various subgroups.

The preferred message content and style focused on clear, fact-based, and honest messages that address immediate side effects and long-term health impacts of the vaccine. This was not surprising given the high prevalence of vaccine hesitancy [54]. Positive, hopeful, and collective (vs. individual) appeal messages were valued, which may elicit a sense of community and responsibility to protect others [42]. The findings suggest messaging about returning to normal/pre-pandemic activities resonated with African American participants and may have motivated vaccination. However, with substantial social distancing recommendations and policies no longer in place, collective appeals or other- (vs. self-) referencing messages that draw on vaccine confidence, benefits, and hope may be important for promoting boosters, especially for those with low perceived risk [55] or who have been frustrated and annoyed about limited immunity and the need for additional boosters [56]. The results show that strong vaccine efficacy and statistical evidence were also helpful to increase hope and initial vaccination intentions, though persuasive messaging about the continued risk of COVID with loss-frames or fear-based appeals about the disease may influence attitudes and promote booster intentions [48, 57, 58].

Mistrust in the vaccine, health care entities, and governmental bodies was common across many studies. Historically, mistrust has also influenced flu vaccination beliefs and behaviors among African Americans [38] and lends itself to longer vaccination deliberation [53]. A trauma-informed strategy acknowledging historical medical mistreatment and structural racism while emphasizing safety and transparency would help align COVID communication with community needs, values, and strengths [59]. Others have emphasized the importance of developing culturally appropriate messages through audience segmentation, listening circles, and community partnerships to co-create communication strategies that will be highly acceptable among African Americans [52, 60–63]. Taking a tailored approach based on audience insights and information needs [64] would also foster collaboration and empower Black communities, both key aspects of trauma-informed care [65], to help identify who should spread information and how to best reach the population with messages that resonate [66, 67].

The nature of vaccine hesitancy is context specific [68, 69]; therefore, this review has some limitations. The credibility and trustworthiness of messengers and information sources were not consistently reported across studies, making them difficult to compare. Despite our focus on African American adults in the USA, we included studies with participants of other ethno-racial identities and were limited to the authors’ reports by identity groups. Finally, the studies focused on the initial roll-out of COVID-19 vaccination, which was likely heavily influenced by the experience of the COVID-19 pandemic and rapid vaccine development; booster vaccination is markedly lower [3] and may require additional segmenting and tailored messaging. For example, although some commonalities regarding sub-populations and geographic locations were discussed, future research should investigate differences by geographic locations and additional subgroups within the Black community, including by vaccination status or hesitancy [70]. However, the insights from this review can inform efforts to encourage initial vaccination, booster vaccination for emerging variants, or other novel vaccines.

The communication strategies identified through this review highlight the importance of partnerships between public health and community-based organizations. Collaborating with Black health care providers and local community leaders who are known and trusted by the target community members on a multimodal communication strategy that uses trauma-informed, fact-based messages with a collective action appeal will be important to address hesitancy and promote vaccine confidence among Black communities.

## Supplementary Information

Below is the link to the electronic supplementary material.

Supplementary file1 (DOCX 20 KB)
