# Supplementary material for: COVID-19 Vaccination Messengers, Communication Channels, and Messages Trusted Among Black Communities in the USA: a Review
Source: J Racial Ethn Health Disparities. 2023 Nov 10;12(1):134–47. doi: 10.1007/s40615-023-01858-1 (PMC11345940; doi:10.1007/s40615-023-01858-1)
Supplement: Supplementary file 1 — Supplementary file1 (DOCX 20 KB) [file 40615_2023_1858_MOESM1_ESM.docx]

| **Supplemental Table. QuADS criteria and scores** | | | | | | | | | | | | | |
| --- | --- | --- | --- | --- | --- | --- | --- | --- | --- | --- | --- | --- | --- |
|  | **QuADS Criteria & Scores** | | | | | | | | | | | | |
| **Study author, year** | **Theoretical/ conceptual underpinning** | **Research aim/s** | **Setting, target population** | **Study design** | **Appropriate sampling** | **Rationale for data collection tool/s** | **Data collection tool/s format, content** | **Description of data collection procedure** | **Recruitment data provided** | **Justification for analytic method** | **Appropriate analytic method** | **Stakeholder consideration** | **Strengths and limitations** |
|  |  |  |  |  |  |  |  |  |  |  |  |  |  |
| Balasuriya et al, 2021 | 3 | 3 | 3 | 3 | 3 | 3 | 3 | 3 | 3 | 3 | 3 | 3 | 3 |
| Bogart et al, 2021 | 3 | 3 | 2 | 3 | 2 | 2 | 3 | 3 | 3 | 3 | 3 | 2 | 3 |
| Burkhardt et al, 2022 | 2 | 3 | 3 | 2 | 2 | 1 | 2 | 3 | 3 | 2 | 2 | 1 | 2 |
| Butler et al, 2022 | 3 | 3 | 3 | 3 | 2 | 3 | 3 | 3 | 3 | 3 | 3 | 3 | 3 |
| Davis et al, 2022 | 2 | 3 | 3 | 3 | 2 | 2 | 3 | 3 | 3 | 2 | 3 | 2 | 2 |
| Dhanani and Franz, 2022 | 3 | 3 | 3 | 2 | 3 | 2 | 3 | 3 | 3 | 2 | 3 | 1 | 2 |
| Dong et al, 2022 | 3 | 3 | 3 | 3 | 3 | 2 | 3 | 3 | 3 | 3 | 3 | 3 | 3 |
| Fisher et al, 2021 | 2 | 3 | 3 | 3 | 3 | 2 | 3 | 2 | 2 | 2 | 3 | 1 | 3 |
| Francis et al, 2021 | 3 | 3 | 3 | 3 | 2 | 2 | 3 | 3 | 3 | 2 | 3 | 1 | 3 |
| Gadarian et al, 2022 | 3 | 3 | 3 | 2 | 3 | 2 | 3 | 3 | 3 | 2 | 2 | 1 | 3 |
| Huang and Green, 2022 | 3 | 3 | 2 | 3 | 2 | 2 | 3 | 2 | 2 | 3 | 3 | 2 | 3 |
| Karpman et al, 2021 | 3 | 3 | 2 | 3 | 2 | 2 | 2 | 3 | 2 | 2 | 3 | 2 | 0 |
| Kerrigan et al, 2022 | 3 | 3 | 3 | 3 | 2 | 2 | 3 | 3 | 3 | 3 | 2 | 3 | 3 |
| Kricorian and Turner, 2021 | 3 | 3 | 3 | 3 | 2 | 2 | 3 | 3 | 2 | 2 | 3 | 2 | 3 |
| Lee Rogers and Powe, 2022 | 3 | 3 | 3 | 3 | 2 | 3 | 3 | 2 | 2 | 3 | 3 | 2 | 1 |
| Lieu et al, 2022 | 3 | 3 | 3 | 3 | 3 | 2 | 3 | 3 | 3 | 2 | 3 | 1 | 2 |
| Majee et al, 2022 | 3 | 3 | 3 | 3 | 3 | 2 | 3 | 3 | 2 | 3 | 3 | 3 | 0 |
| Momplaisir et al, 2021 | 3 | 3 | 3 | 3 | 3 | 2 | 2 | 3 | 2 | 2 | 3 | 2 | 3 |
| Osakwe et al, 2021 | 2 | 3 | 3 | 3 | 3 | 2 | 3 | 3 | 3 | 3 | 3 | 2 | 3 |
| Redmond et al, 2022 | 3 | 3 | 3 | 3 | 2 | 3 | 3 | 3 | 3 | 3 | 3 | 3 | 3 |
| Sekimitsu et al, 2022 | 3 | 3 | 3 | 2 | 2 | 2 | 3 | 2 | 3 | 2 | 3 | 3 | 3 |
| Zhou et al, 2022 | 3 | 3 | 3 | 3 | 2 | 2 | 3 | 3 | 3 | 3 | 3 | 3 | 3 |
| *Notes:* QuADS, Quality assessment with diverse studies (Harrison et al, 2021); Scores 0 no mention at all to 3 explicit details provided | | | | | | | | | | | | | |

Rabin Y and Kohler RE. COVID-19 Vaccination Messengers, Communication Channels, and Messages Trusted among Black Communities in the United States: A Review. Journal of Racial and Ethnic Health Disparities
